# Luteolin reversed anxiety and depressive-like behavior via modulation of the NF-κB/NLRP3 inflammasome axis in the hippocampus of rats subjected to sleep deprivation

**DOI:** 10.22038/IJBMS.2024.75068.16277

**Published:** 2024

**Authors:** Fang Xiong, Xuewen Lv

**Affiliations:** 1 Department of Sleep Disorders and Neuroses, Brain Hospital of Hunan Province (The Second People’s Hospital of Hunan Province), No. 427, Section 3, Furong Middle Road, Changsha, 410000, China; 2 Department of Critical Medicine, Brain Hospital of Hunan Province (The Second People’s Hospital of Hunan Province), No. 427, Section 3, Furong Middle Road, Changsha, 410000, China

**Keywords:** Anxiety, Depression, Hippocampus, Luteolin, Neuroinflammation, Sleep deprivation

## Abstract

**Objective(s):**

In this study, we assessed the impact of luteolin (LUT) on mood disorders (specifically anxiety and depression) induced by sleep deprivation (SD) by regulating pathways associated with neuroinflammation.

**Materials and Methods:**

Rapid eye movement (REM) SD was employed to induce anxiety and depression in the animal subjects. The animals were treated with PAX (15 mg/kg, positive control) and LUT (10 and 20 mg/kg) for a duration of 21 days. The anxiety and depressive disorders were evaluated using behavioral tests. Following the sacrifice of the animals, hippocampal tissues were stored for molecular investigations.

**Results:**

SD resulted in anxiety, as evidenced by the elevated plus maze test and open field test. Furthermore, the findings from the sucrose performance test, forced swimming test, and tail suspension test confirmed the presence of depressive-like behaviors in the animals. The nuclear factor kappa B (NF-κB) and NLR family pyrin domain containing 3 (NLRP3) inflammasome components, including apoptosis-associated speck-like protein containing a C-terminal caspase recruitment domain (ASC), NLRP3, and active Caspase-1, were up-regulated in the hippocampus (HC) of the animals subjected to REM SD. However, treatment with LUT demonstrated a significant reversal of the behavioral changes by modulating the NF-κB and NLRP3 inflammasome components in the HC.

**Conclusion:**

It can be concluded that LUT demonstrated antidepressant effects via regulation of the NF-κB/NLRP3 inflammasome axis components in the HC.

## Introduction

Sleep deprivation (SD), which refers to irregular and insufficient sleep, is a prevalent and significant issue in our modern society (1). Numerous studies have indicated that chronic disruption of sleep can lead to pathological mood disorders (i.e., anxiety and depression) in humans (2, 3). Rapid eye movement (REM) sleep is a sleep phase distinguished by vivid dreaming, swift eye movements, and increased brain activity. REM sleep plays a vital role in regulating mood stability and preventing mood disorders such as depression and anxiety (4). In fact, more than 90% of individuals with depression report experiencing impairments in sleep quality (5). As a result, sleep disturbances are considered risk factors for the development of depression (6). Several reports suggest that long-term sleep disturbances can cause changes in the brain that resemble those observed in individuals with depression (7, 8). However, there is still limited understanding of the specific mechanisms that link inadequate sleep and depression. One possible mechanism suggests that a lack of adequate sleep could potentially contribute to depression by impacting the signaling pathways responsible for synaptic plasticity in the hippocampus (HC)(9).

 A recent study demonstrated that inflammation can lead to phenotypes similar to depression by causing changes in brain-derived neurotrophic factor (BDNF), a critical protein involved in synaptic plasticity in the prefrontal cortex, HC, and nucleus accumbens (10). SD adversely impacts the function of the HC, a vital brain region involved in regulating mood and behavior (11). Hippocampal neurons are particularly susceptible to damage in the early stages of neuropsychiatric disorders (12, 13). Emerging research suggests that neuroinflammation occurring in the HC plays a substantial role in the manifestation of symptoms associated with anxiety and depression (14, 15). Neuroinflammation is identified by the active microglia and astrocytes, the release of chemokines and proinflammatory cytokines, as well as the activation and infiltration of leukocytes (16). 

The release of proinflammatory cytokines is associated with the activation of multi-protein complexes in the cytosol known as inflammasomes. Among the various inflammasomes, the NLR family pyrin domain-containing 3 (NLRP3) inflammasome plays a crucial role in the pathophysiology of depression (17-19). This inflammasome consists of a sensor molecule, NLRP3, an adaptor protein called apoptosis-associated speck-like protein (ASC), and an effector molecule known as Caspase-1 (Casp-1). Upon activation, the NLRP3 inflammasome in microglia is responsible for the cleavage and activation of interleukin-1β (IL-1β) and IL-18 through Casp-1-mediated processing (20). The structure of the NLRP3 inflammasome reveals that ASC binds both Casp-1 and NLRP3, functioning to assemble the NLRP3 inflammasome. This assembly ultimately leads to the activation of Casp-1 and the subsequent release of mature proinflammatory cytokines (21).

Luteolin (LUT) naturally occurs as a glycosylated form and can be found in various fruits and vegetables such as pepper, broccoli, thyme, apple skins, and celery (22). Several scientific investigations have been conducted to explore the pharmacological properties of LUT, a compound of interest. These studies have collectively demonstrated its wide-ranging effects, including anti-inflammatory, antioxidant, and neuroprotective characteristics (23, 24). Additionally, it has been shown to have the ability to penetrate the blood-brain barrier (BBB)(25). Furthermore, LUT has shown promising neuroprotective effects, which may be attributed to its ability to regulate molecular signaling cascades involved in neuronal health and function (26). Recent research has indicated that LUT exhibits anxiolytic- and antidepressant-like effects, which are mainly attributed to its anti-inflammatory effects and protect the nervous system (27-29). However, the precise mechanisms responsible for the anxiolytic- and antidepressant effects of LUT remain unclear, necessitating further investigation in order to gain a comprehensive understanding of its pharmacological actions.

In this study, we aimed to assess the anxiolytic- and antidepressant-like effects of LUT in a rat model of REM SD. To achieve this objective, we conducted an experiment to investigate two main aspects: (1) the manifestation of anxiety- and depressive-like behaviors in the animals, and (2) the levels of components related to the NF-κB/NLRP3 inflammasome axis in the HC.

## Materials and Methods

***Animals***

Forty Wistar male rats, aged three months and weighing between 150 and 220 g, were housed in the animal facility at Hunan University of Chinese Medicine. The rats were maintained under standard temperature conditions of 22±2 ^°^C and subjected to a 12/12 hr light/dark cycle, with lights on from 08:00 to 20:00. Throughout the study, the animals were provided with unrestricted access to standard food and water. The research protocol was reviewed and approved by the Ethics Committee of Brains Hospital of Hunan Province, with the ethical code 2021023, in accordance with the guidelines outlined in the Care and Use of Laboratory Animals (8^th^ edition).

***Experimental design***

A total of 40 rats were randomly assigned to the study groups, with eight animals per group. The groups were as follows: 1) Control (Co), consisting of healthy animals that received a solvent; 2) SD, comprising model animals that also received a solvent; 3) SD+LUT10, consisting of model animals that were administered 10 mg/kg of LUT; 4) SD+LUT20, comprising model animals that received 20 mg/kg of LUT; and 5) PAX, consisting of model animals that received 15 mg/kg of paroxetine hydrochloride (PAX, positive control). The administration of the solvent, LUT, or PAX was performed through intraperitoneal injections over a period of three weeks. The dosages of LUT and PAX were determined based on the findings of a previous study (30). For administration of LUT, a solvent consisting of PBS with 0.1% DMSO (dimethyl sulfoxide) from Sigma-Aldrich was utilized. PAX, on the other hand, was dissolved in a 0.9% saline solution

***Sleep deprivation model***

The REM SD model in rats was established through the utilization of a modified multi-platform method (31). The rat boxes used for REM SD had dimensions of 120 cm in length, 60 cm in width, and 40 cm in height. Inside the boxes, there were six platforms spaced 10 cm apart, allowing the rats to freely jump from one platform to another. The platforms were surrounded by water maintained at a temperature of 24-26 ^°^C. The rats had the freedom to drink, eat, and move around on the platforms. The distance between the water surface and the platforms was approximately 1 cm. By preventing the rats from entering REM sleep, the sleep-deprivation chamber ensured that their muscle tone remained active, preventing them from falling into a deep sleep. REM SD was maintained for a period of 72 hr. 

***Behavioral tests***

To assess potential alterations in behavior associated with depression and anxiety, a comprehensive set of tests was employed. These tests included the sucrose preference test (SPT), which measures anhedonia and is commonly used as an indicator of depressive-like behavior in animal models. Additionally, the forced swimming test (FST) and tail suspension test (TST) were conducted to evaluate despair-like behavior, which is often associated with depression. Furthermore, the open field test (OFT) and elevated-plus maze (EPM) test were employed to assess anxiety-like behavior. Independent experimenters who were not aware of the treatment schedule conducted these tests. Importantly, all of the behavior tests were performed during the light period from 8:00 a.m. to 11:00 a.m. This period was chosen to ensure consistency in the environmental conditions under which the tests were conducted.

*SPT*

The main objective of SPT is to evaluate anhedonia, which serves as an indicator of diminished interest. SPT was carried out following a previously established protocol (32). In brief, prior to SPT, all rats were acquainted with a 1% sucrose solution (w/v). For this purpose, two bottles containing the 1% sucrose solution were placed in each cage to allow the rats to become familiar with the taste. After depriving the rats of water for a duration of 14 hr, one of the bottles holding the 1% sucrose solution was substituted with tap water. SPT was performed on rats that were kept in individual cages. These rats had unlimited access to two bottles: one bottle contained a 1% sucrose solution (100 ml), while the other bottle contained tap water (100 ml). After a duration of 1 hr, the percentage of sucrose consumed relative to the total liquid intake (sucrose consumed+water consumed) was calculated as the sucrose preference.

*FST*

An 80 cm tall and 30 cm diameter glass cylinder was filled with 40 cm of water at a temperature of 25 ^°^C. The experimental setup was employed to evaluate the behavioral responses of the rats in two consecutive sessions, specifically referred to as the training and main test sessions. In the training session, each rat was introduced into the cylinder and required to engage in swimming activity for a period of 10 min. This allowed for the assessment of the rats’ behavior and performance in the swimming task under controlled conditions. After a 24-hour interval, the same procedure was repeated, but this time the test session lasted for 5 min. The behavior of the animals in this session was recorded on video by an observer who was unaware of the experiment, and the durations of immobility, swimming, and climbing (the vertical movement of the forepaws directed towards the sides of the swim cylinder walls) were measured and analyzed (33). A duration of motionlessness, swimming, and climbing was measured, with immobility being regarded as an indicator of depressive disorder.

*TST*

One day following the FST, the TST was conducted to assess behaviors associated with depression. In this test, animals were hung above a solid surface by their tail using vinyl tape to a box for a total of 6 min. The allotted time was divided into a 2-minute period for adaptation and a 4-minute duration for the primary test. The duration of immobility was documented (34).

*OFT*

OFT was conducted to assess anxiety-like behavior, as documented in previous studies (35). The apparatus (100×100×40 cm3) comprises a floor divided into 25 identical squares. Each animal was individually positioned at the central region of the open field and allowed 5 min to freely explore, while their behavior was meticulously documented. The duration of immobility was assessed over a period of 5 min. Following the completion of the test, the rat was returned to its home cage by the experimenter. To remove any olfactory cues, the apparatus was cleaned with 90% ethanol after each test. 

*EPM*

The EPM test was utilized to assess anxiety levels in each individual rat. The maze apparatus comprises four arms, with two arms being open (50×10 cm2) and the remaining two arms being closed (50×10×40 cm3). The apparatus was positioned 50 cm above the floor. In the experiment, every animal was positioned in the central area of the maze and given the freedom to move around. A camera recorded the movements of the rats for a period of 5 min, during which the duration spent in both the open arms and number of open-arm entries were documented (36). 

***Tissue preparation***

Upon completion of the experiment, the rat brains were utilized for molecular studies, specifically involving 4 brains for quantitative reverse transcription polymerase chain reaction (qRT-PCR) and another set of 4 brains for enzyme-linked immunosorbent assay (ELISA). Subsequent to the rats’ sacrifice, fresh samples of the HC were promptly extracted, carefully placed into freezing tubes, and preserved at an ultra-low temperature of -80 ^°^C.

***qRT-PCR***

The fresh samples were subjected to a quantitative reverse transcription polymerase chain reaction (qRT-PCR) test to assess the levels of gene expression of various inflammatory components. These components included NF-κB, ASC, NLRP3, and active Casp-1in the HC of animals. The total RNA was extracted using the Tripure Isolation Reagent (Roche Applied Science). The purity of the extracted RNA was assessed using the NanoDrop™ spectrophotometer (Thermo Fisher Scientific). For reverse transcription, the PrimeScript RT Reagent Kit (Takara Bio Inc.) was utilized to synthesize complementary DNA. The StepOnePlus™ Real-Time PCR System (Applied BioSystems, Foster City, CA, USA) was employed to perform qRT-PCR. In order to standardize the gene expression measurements, beta-actin (β-actin) was utilized as a reference gene. The 2^-ΔΔCt^ formula as a relative quantification strategy was employed to calculate the fold change in gene expression. This method involves comparing the cycle threshold (Ct) values of the target gene with those of the reference gene. This method allows for the quantification of changes in gene expression levels between different experimental conditions (37). The specific sequences of the primers for each gene can be found in Table 1.

***ELISA***

The ELISA kit was used to estimate hippocampal levels of NF-κB, NLRP3 (BioSource), ASC (BioSource), and Casp-1 (Cusabio). Briefly, HC was extracted from the brain of each animal and homogenized by lysis buffer containing NaCl, Igepal (1%), Tris–HCl (pH 8.0), glycerol (10%), sodium vanadate, EDTA, PMSF, and EGTA. The homogenate was centrifuged (20,000 rpm at 4 ^°^C for 15 min). Then, the supernatant was separated and utilized for ELISA. ELISA was performed in accordance with manufacturers’ guides. Fifty microliters of supernatants were incubated in the ELISA 96-well plate pre-coated by monoclonal antibodies. After blocking, the horseradish peroxidase (HRP)-labeled detection antibody was added. The measurement of the color reaction was quantified using a plate reader set to a wavelength of 450 nm (38). 

***Data analysis***

The data was processed using IBM® SPSS® version 23.0 statistical software and the results were reported in terms of the Means±SD (standard deviation). One-way analysis of variance (ANOVA) was conducted to assess the presence of any significant differences between the means of the variables being studied. Subsequently, the Tukey test was employed as a *post hoc* test to further investigate pairwise differences. In order to determine statistical significance, a *P*-value below 0.05 was considered to be the threshold.

## Results

***Behavioral tests***

*SPF*

**Figure 1:**
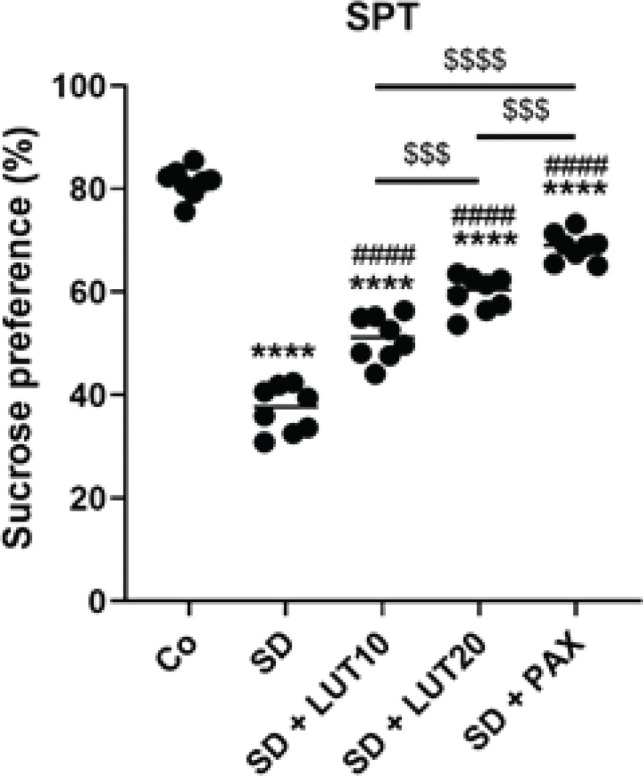
Impacts of luteolin (LUT) on depressive phenotypes (TST) in Rapid eye movement (REM) SD-induced rats

The results of the SPT demonstrated that the animals exposed to SD exhibited a significant reduction in sucrose consumption relative to the Co group (*P*<0.0001, Figure 1). However, administration of both LUT at doses of 10 and 20 mg/kg, as well as PAX (15 mg/kg), caused a significant improvement in sucrose performance relative to the SD group. These findings suggest that LUT, similar to PAX, has the potential to effectively enhance sucrose preference in the SPT. 

*FST*

**Figure 2:**
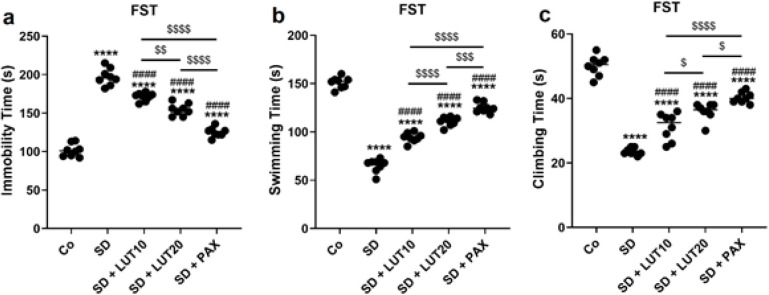
Impacts of luteolin (LUT) on depressive phenotypes (FST) in REM SD-induced rats

The results obtained from the FST demonstrated that exposure of animals to SD led to a significant increase in immobility time *(P*<0.0001, Figure 2a), as well as a reduction in swimming time (*P*<0.0001, Figure 2b) and climbing time (*P*<0.0001, Figure 2c) relative to the Co group. However, administration of both LUT at doses of 10 and 20 mg/kg, as well as PAX at a dose of 15 mg/kg, resulted in a significant improvement in immobility time, swimming time, and climbing time when relative to the SD group. These findings suggest that LUT, similar to PAX, demonstrated comparable efficacy in improving the depressive-like behavior observed in the FST.

*TST*

**Figure 3:**
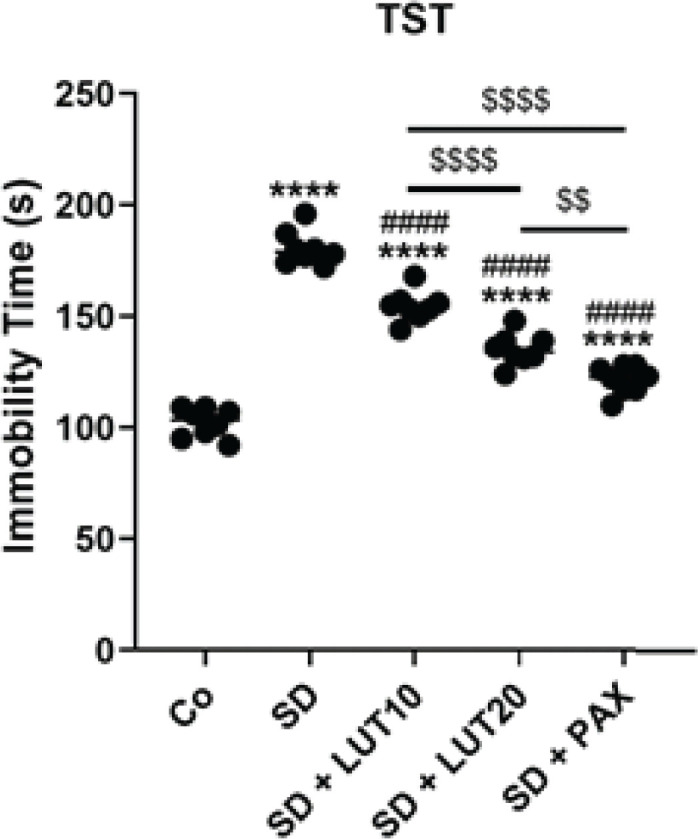
Impacts of luteolin (LUT) on depressive phenotypes (TST) in REM SD-induced rats

The results obtained from the TST revealed that when animals were subjected to SD, there was a notable increase in immobility time (*P*<0.0001, Figure 3) relative to that of Co group. However, administration of both LUT at doses of 10 and 20 mg/kg, as well as PAX at a dose of 15 mg/kg, resulted in a significant improvement in immobility time in the treatment groups relative to the SD group. These findings indicate that LUT showed comparable results to PAX in terms of its ability to improve the enhanced immobility observed in the TST due to SD.

*OFT*

**Figure 4:**
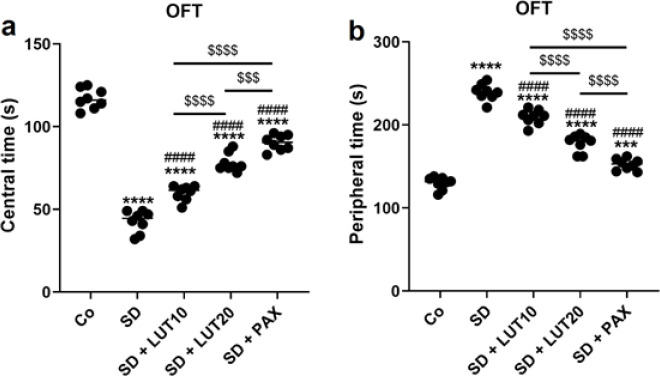
Impacts of luteolin (LUT) on anxiety phenotypes (OFT) in REM SD-induced rats

The findings derived from the OFT revealed significant changes in central and peripheral time when animals were subjected to REM SD. Specifically, there was a noticeable decrease in time spent in the central area (*P*<0.0001, Figure 4a) and an increase in the time spent in the peripheral area (*P*<0.0001, Figure 4b) compared to the control (Co) group. However, administration of both LUT at doses of 10 and 20 mg/kg, as well as PAX at a dose of 15 mg/kg, led to a significant modulation of central and peripheral time in the treatment groups relative to the SD group. These findings indicate that LUT demonstrated effects comparable to PAX in terms of its ability to modify the alterations observed in central and peripheral time resulting from REM SD. The study provides valuable insights into the potential therapeutic benefits of LUT in ameliorating the behavioral changes associated with SD.

*EPM*

**Figure 5:**
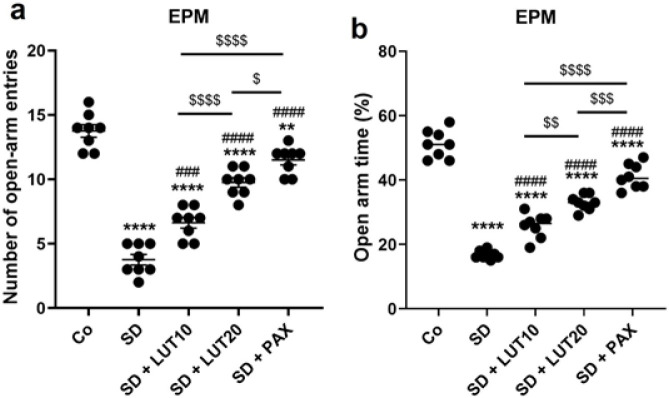
Impacts of luteolin (LUT) on anxiety phenotypes (EPM) in REM SD-induced rats

The findings from the EPM test revealed that when animals were subjected to REM SD, there was a reduction in the number of open-arm entries (*P*<0.0001, Figure 5a) and the time spent on the open arm (*P*<0.0001, Figure 5b) relative to the Co group. However, administration of both LUT at doses of 10 and 20 mg/kg, as well as PAX at a dose of 15 mg/kg, resulted in a significant improvement in the number of open-arm entries and time spent on the open arm in the treatment groups relative to the SD group. These findings suggest that LUT exhibited comparable results to PAX in terms of its ability to improve the changes observed in the number of open-arm entries and the open arm due to REM SD.

***qRT-PCR***

**Figure 6:**
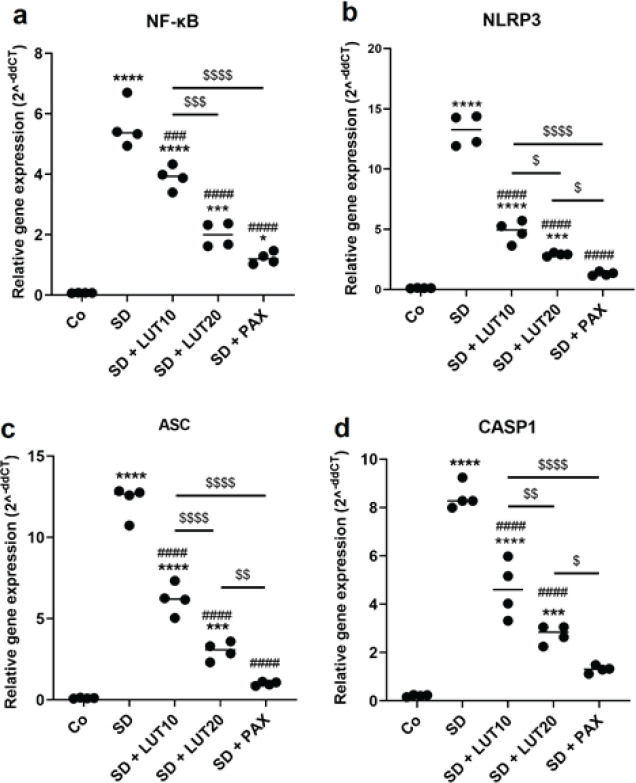
Impacts of luteolin (LUT) on the gene expression of NF-κB/NLRP3 inflammasome axis components in the HC of REM SD-induced rats

This study aimed to evaluate the mean expression levels of NF-κB, ASC, NLRP3, and active Casp-1 genes in the hippocampus region of animals exposed to REM SD. The results revealed a significant up-regulation in the expression of hippocampal NF-κB, ASC, NLRP3, and active Casp-1 in the SD group relative to the Co group (*P*<0.000001, Figure 6a-d). Interestingly, the SD+LUT10, SD+LUT20, and SD+PAX groups exhibited a significant reduction in the expression of hippocampal NF-κB, ASC, NLRP3, and Casp-1 relative to those of the SD group. These findings suggest that LUT demonstrated comparable effects to PAX in terms of attenuating the elevated gene expression of hippocampal NF-κB, NLRP3, ASC, and Casp-1 induced by REM SD. The study provides important insights into the potential therapeutic benefits of LUT in mitigating the detrimental effects of SD.

***ELISA***

**Figure 7:**
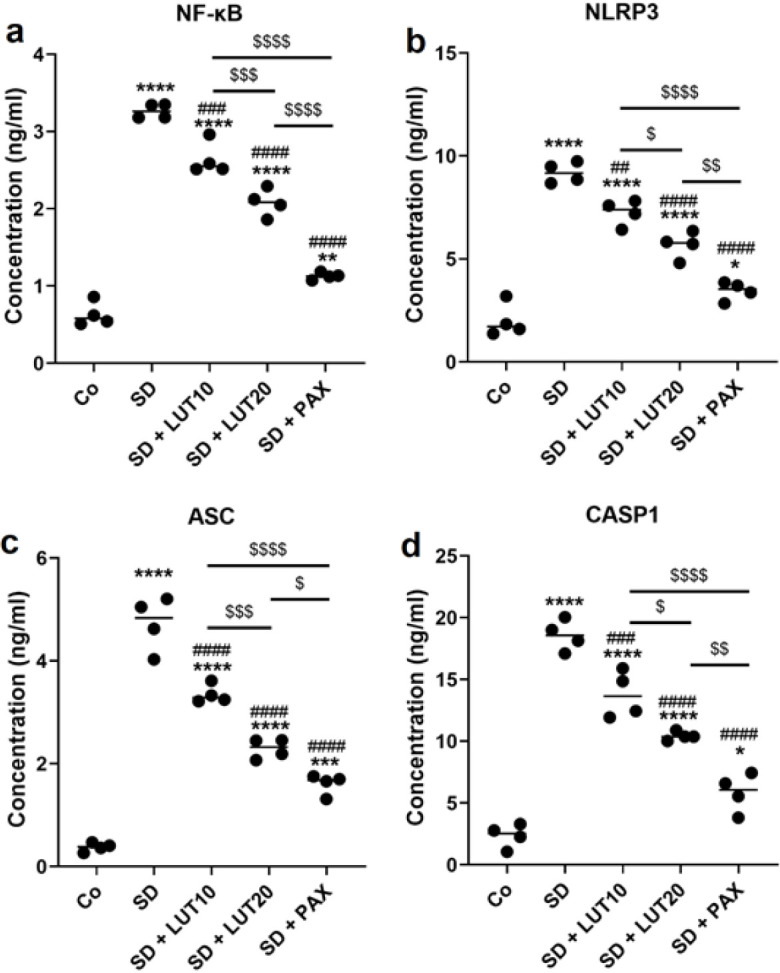
**.** Impacts of luteolin (LUT) on the protein levels of NF-κB/NLRP3 inflammasome axis components in the HC of REM SD-induced rats

The data obtained from the study revealed a significant elevation in the protein levels of NF-κB, NLRP3, ASC, and active Casp-1 in the hippocampus of animals exposed to SD relative to the Co group (*P*<0.000001, Figure 7a-d). Conversely, the protein levels of hippocampal NF-κB, NLRP3, ASC, and Casp-1 were significantly reduced in the SD+LUT10, SD+LUT20, and SD+PAX groups relative to the SD group. These findings provide evidence that LUT demonstrated comparable efficacy to PAX in terms of its ability to decrease the protein levels of hippocampal NF-κB, ASC, NLRP3, and active Casp-1, which were elevated due to REM SD. The study highlights the potential of LUT as a therapeutic intervention for mitigating the detrimental effects of SD on hippocampal protein expression.

**Table 1:** Sequences of the primers used for qPCR

**Gene**	**Forward primer**	**Reverse Primer**
NF-κB	AATTGCCCGGGGGCAAT	TCCCGTAATCCGGCGTAA
NLRP3	GGAGTGGCCGGGTTTGCTGG	GGTGTAGCCTCTGTTGAGGT
Caspase-1	GTGGAGAGTTTGAAGGAGTGGT	GATGAGATGCTGAATGAAGAGG
ASC	TCTCCAGGGGTATGGGGTGG	GAGTGCAACTGTGTGTTGGT
b-actin	ACAACCAAGTTGCAGCTCCTC	CTGACCCATAGGGACCATCAC

Immobility time in FST; ^****^P<0.0001 relative to the Co group, ^####^*P<*0.0001 relative to the SD group, ^$$^*P<*0.0001 and ^$$$$^*P<*0.000001 between treatment groups, Co: Normal and healthy rats received vehicle, SD: model animals received vehicle, SD+LUT10: model animals received 10 mg/kg LUT, SD+LUT20: model animals received 20 mg/kg LUT, and SD+PAX: model animals received 15 mg/kg PAX

FST: forced swimming test; Co: Control; SD: Sleep deprivation

a. Immobility duration (s), c. Swimming duration (s), and c. Climbing duration (s); ^****^P<0.0001 relative to the Co group, ^####^*P<*0.0001 relative to the SD group, ^$^*P<*0.0005, ^$$^*P<*0.0001, ^$$$^*P<*0.001, and ^$$$$^*P<*0.000001 between treatment groups, Co: Normal and healthy rats received vehicle, SD: model animals received vehicle, SD+LUT10: model animals received 10 mg/kg LUT, SD+LUT20: model animals received 20 mg/kg LUT, and SD+PAX: model animals received 15 mg/kg PAX

FST: forced swimming test; Co: Control; SD: Sleep deprivation; REM: Rapid eye movement

Immobility time in FST; ^****^P<0.0001 relative to the Co group, ^####^*P<*0.0001 relative to the SD group, ^$$^*P<*0.0001 and ^$$$$^*P<*0.000001 between treatment groups, Co: Normal and healthy rats received vehicle, SD: model animals received vehicle, SD+LUT10: model animals received 10 mg/kg LUT, SD+LUT20: model animals received 20 mg/kg LUT, and SD+PAX: model animals received 15 mg/kg PAX

TST: Tail suspension test; Co: Control; SD: Sleep deprivation; REM: Rapid eye movement

a. Duration spent in the central area (s) and b. Duration spent in the central area. ^****^P<0.0001 relative to the Co group, ^####^*P<*0.0001 relative to the SD group, ^$$$^*P<*0.001 and ^$$$$^*P<*0.000001 between treatment groups, Co: Normal and healthy rats received vehicle, SD: model animals received vehicle, SD+LUT10: model animals received 10 mg/kg LUT, SD+LUT20: model animals received 20 mg/kg LUT, and SD+PAX: model animals received 15 mg/kg PAX

OFT: Open field test; Co: Control; SD: Sleep deprivation; REM: Rapid eye movement

a. Number of open-arm entries and b. Open arm time (%). ^****^P<0.0001 relative to the Co group, ^####^*P<*0.0001 relative to the SD group, $ *P<*0.0005, ^$$^*P<*0.0001, ^$$$^*P<*0.001, and ^$$$$^*P<*0.000001 between treatment groups Co: Normal and healthy rats received vehicle, SD: model animals received vehicle, SD+LUT10: model animals received 10 mg/kg LUT, SD+LUT20: model animals received 20 mg/kg LUT, and SD+PAX: model animals received 15 mg/kg PAX

EPM: Elevated-plus maze; Co: Control; SD: Sleep deprivation; REM: Rapid eye movement

a. NF-κB, b. NLRP3, c. ASC, and d. active Casp-1. ^***^P<0.001 and ^****^P<0.0001 relative to the Co group, ^###^*P<*0.0001 and ^####^*P<*0.0001 relative to the SD group, ^$^*P<*0.0005, ^$$^*P<*0.0001, ^$$$^*P<*0.001, and ^$$$$^*P<*0.000001 between treatment groups, Co: Normal and healthy rats received vehicle, SD: model animals received vehicle, SD+LUT10: model animals received 10 mg/kg LUT , SD+LUT20: model animals received 20 mg/kg LUT , and SD+PAX: model animals received 15 mg/kg PAX

HC: hippocampus; ASC: apoptosis-associated speck-like protein; Co: Control; SD: Sleep deprivation; REM: Rapid eye movement

a. NF-κB, b. NLRP3, c. ASC, and d. active Casp-1. ^***^P<0.001 and ^****^P<0.0001 relative to the Co group, ^###^*P<*0.0001 and ^####^*P<*0.0001 relative to the SD group, ^$^*P<*0.0005, ^$$^*P<*0.0001, ^$$$^*P<*0.001, and ^$$$$^*P<*0.000001 between treatment groups, Co: Normal and healthy rats received vehicle, SD: model animals received vehicle, SD+LUT10: model animals received 10 mg/kg LUT , SD+LUT20: model animals received 20 mg/kg LUT , and SD+PAX: model animals received 15 mg/kg PAX

HC: hippocampus; Co: Control; SD: Sleep deprivation; REM: Rapid eye movement

## Discussion

In this study, we employed REM SD to induce behavioral alterations, and subsequently administered UT (10 and 20 mg/kg) and PAX (15 mg/kg) to mitigate these alterations. The findings of our investigation demonstrated that administration of both LUT and PAX effectively ameliorated anxiety and depression-like behaviors induced by SD. Furthermore, both LUT and PAX demonstrated the ability to alleviate the activity of the inflammasome pathway by down-regulation of genes involved in neuroinflammation, including NF-κB, NLRP3, ASC, and active Casp-1 in the HC. Our study demonstrates that LUT and PAX exhibited comparable results in terms of alleviating the anxiety and depressive symptoms induced by REM SD.

The results of our study suggest that subjecting animals to 72 hr of REM SD led to the development of anxiety and depressive phenotypes. These findings were supported by various behavioral tests conducted during the study. We observed a decrease in sucrose intake, increased immobility during the TST, reduced swimming and climbing behavior, and an increase in immobility during the FST, all of which are indicators of depressive behaviors in the animals. Additionally, anxiety-like phenotypes were confirmed by an increase in the duration spent in the peripheral zone and a decrease in the duration spent in the central zone of the OFT. Additionally, there was a reduction in the number of open-arm entries and the duration spent in the open arm of the elevated maze paradigm (EMP), which offers additional substantiation of anxiety-like behaviors exhibited by the animals. Numerous studies have corroborated our research outcomes. In line with our findings, anxiogenic behaviors were evident not only in male mice subjected to 72 hr of REM SD in the EPM-M1 mice test, and female BALB/c mice deprived of REM sleep for 48 hr, but also in male Wistar rats that experienced REM SD for a continuous period of 21 days, with each day comprising 18 hr of deprivation (39, 40). In addition, rats and mice that underwent REM SD for a duration of 24 hr displayed anxiety-like behaviors (41-43). During the SPT, male Wistar rats that experienced a REM SD period of 48 hr exhibited a decrease in their intake of sucrose relative to the control rats (44). In the FST, which serves as a measure of depressive behavior, an augmentation in swimming activity was noted following a 24-hour period of REM SD (45). The initiation of REM sleep is distinguished by a rapid decline in the levels of monoamines, including serotonin, norepinephrine, and dopamine, alongside a concomitant rise in cholinergic activity. This empirical evidence strongly indicates that REM SD can potentially influence emotional states, leading to the development of anxiety and depression (4). Based on the provided information, there is evidence suggesting a link between REM SD and the development of anxiety and depression. The results of the study, along with other studies, indicate that subjecting animals to REM SD leads to the manifestation of anxiety and depression.

The results of our study revealed that exposing animals to 72 hr of REM SD led to a notable elevation in the levels of NF-κB, ACs, NLRP3, and Casp1 in the HC. These findings indicate that the stressor-induced neuroinflammation in the HC is linked to behavioral changes. Anxiety and depression are psychiatric conditions that are associated with a range of pathological processes, one of which is the occurrence of neural cell death in the HC (46, 47). The decrease in hippocampal volume observed in individuals with depressive disorders is associated with neural loss in the HC (48). Existing literature indicates that psychological or physical stressors can trigger the activation of inflammatory pathways, leading to the production of inflammatory cytokines. This cascade of events subsequently leads to both functional and structural alterations in neurons (49). The interaction between the central nervous system (CNS) and the immune system is of utmost importance in the context of stress-induced neuroinflammation and its association with anxiety and depression (50). Anxiety and depressive-like behaviors have been found to be linked to elevated levels of cytokines, such as TNF-α, in both the HC and striatum (51). Among the diverse array of cytokines, IL-1β appears to exert a significant influence on the pathological manifestations of stress-induced depressive-like behavior (52). IL-1β has the ability to decrease the formation of new neurons, known as neurogenesis, in hippocampal progenitor cells by stimulating the kynurenine cascade. This phenomenon is commonly observed in individuals with depression. Notably, both inhibitors of this pathway and conventional antidepressant medications have the ability to regulate or modify this effect (53). The NLRP3 inflammasome plays a crucial role as a primary mediator between cells in the maturation and release of inflammatory factors, such as IL-1β (54). The NF-κB/NLRP3 axis has been confirmed to be activated in both depression models and individuals diagnosed with depression (55-57). These findings indicate that the NF-κB/NLRP3 inflammasome pathway can potentially act as a crucial connection between the immune system and the onset of depressive disorders in response to stressors. Stimulation of NF-κB leads to the generation of precursor forms of IL-18 and IL-1β, as well as the NLRP3 protein, through transcriptional processes (58). In diabetic mice, the NLRP3 inflammasome is implicated in the occurrence of pyroptosis and apoptosis in hippocampal neurons, which in turn contribute to the manifestation of depressive-like behavior (59). Therefore, targeting NF-κB/NLRP3 through potential anti-inflammatory components may potentially reduce anxiety and depressive disorders.

In our current study, we administered LUT (10 and 20 mg/kg) and PAX (15 mg/kg) for a duration of three weeks following the exposure of animals to 72 hr of REM SD. When relative to PAX, administration of LUT demonstrated the ability to improve anxiety and depressive-like behaviors in the animals. The results obtained from our study indicate that both LUT and PAX effectively reduced anxiety and depressive disorders in animals subjected to 72 hr of REM SD. These findings were supported by various behavioral tests. We observed an increase in sucrose intake, a decrease in immobility during the TST, increased swimming and climbing behavior, and a decrease in immobility during the FST. Furthermore, anxiety-like disorders were alleviated, as evidenced by a reduction in the duration spent in the peripheral zone and an increase in the duration spent in the central zone of the OFT. Additionally, there was an elevation in the number of open-arm entries and time spent in the open arm of the EMP. The outcomes of our study revealed that the anxiolytic- and antidepressant-like impacts of LUT were linked to the regulation of NF-κB/NLRP3 components in the HC. LUT, a naturally occurring flavonoid abundantly present in a variety of fruits and vegetables, has been extensively investigated for its potential neuroprotective, anxiolytic, and antidepressant properties (23). Recent evidence has confirmed the ability of peripherally administered LUT to penetrate the BBB (60). Research suggests that LUT possesses antidepressant-like qualities by influencing various neurochemical pathways involved in the regulation of mood. Specifically, studies have demonstrated that LUT exerts anxiolytic- and antidepressant effects through its modulation of the gamma-aminobutyric acid (GABA) system, a critical regulator of anxiety and depression (61). Animal studies have further shown that LUT administration can alleviate depressive disorders by elevating levels of neurotransmitters such as serotonin and norepinephrine, which play vital roles in mood regulation (62). Moreover, LUT has been found to possess antioxidant and anti-inflammatory properties, which may contribute to its antidepressant effects by reducing oxidative stress and neuroinflammatory biomarkers (63). More recently, LUT has been shown to exhibit anxiolytic-like and antidepressant-like properties in the HC and prefrontal cortex, attributed to its anti-inflammatory, antioxidant, and neuroprotective properties. In these regions, LUT modulates the expression of NF-κB, NLRP3, IL-1β, IL-18, IL-6, and TNF-α (27). 

## Conclusion

In our study, we discovered that when animals were exposed to 72 hr of REM SD, they exhibited anxiety and depressive-like behaviors. These behaviors were primarily triggered by the activation of the NF-κB/NLRP3 inflammasome axis in the HC. Our findings showed that both doses of LUT (10 and 20 mg/kg) effectively reduced anxiety and depressive disorders and regulated the expression of genes and proteins associated with the NF-κB/NLRP3 pathway in the HC. This suggests that LUT has anxiolytic and antidepressant effects by modulating the NF-κB/NLRP3 inflammasome pathways. Thus, our results provide further evidence supporting the potential use of LUT in the treatment of anxiety and depressive disorders. Additionally, the comparable outcomes between LUT and PAX highlight LUT as a promising alternative or supplementary treatment option to conventional antidepressants.

## Authors’ Contributions

Both authors made significant contributions to the study, including its conception and design. They both actively participated in conducting the experiment, analyzing the data, and either drafting or critically revising the article. Furthermore, both authors equally provided their final approval for the publication of the article. They engaged in thorough discussions about the results and contributed equally to the final manuscript.

## Funding

This study was funded by Brains Hospital of Hunan Province, Hunan, China. 

## Availability of data and material

Data are available from the authors upon reasonable request.

## Ethics Approval

Animals had *ad libitum* access to standard food and water. The present research was approved by the Brains Hospital of Hunan Province Ethics Committee (ethical code: 2021023; Changsha, China) and performed based on the guidelines for the Care and Use of Laboratory Animals (8^th^ edition, National Academies Press).

## Conflicts of Interest

The authors declare that they have no competing interests.
